# The effects of different surface contaminators on the shear bond strength of a universal adhesive system to dentin: an experimental study

**DOI:** 10.34172/joddd.2021.014

**Published:** 2021-05-05

**Authors:** Mahmoud Bahari, Siavash Savadi Oskoee, Mohammad Esmaeel Ebrahimi Chaharom, Nasim Molayi

**Affiliations:** ^1^Department of Operative Dentistry, Dental Faculty, Tabriz University of Medical Sciences, Tabriz, Iran; ^2^Dental and Periodontal Research Center, Dental Faculty, Tabriz University of Medical Sciences, Tabriz, Iran

**Keywords:** Adhesives, Blood, Caries detector, Dental bonding, Haemostatics, Saliva

## Abstract

**Background.** Contamination of dentin surface is one of the common problems in restorative dentistry. The aim was to investigate the effects of different surface contaminators on the dentin shear bond strength (SBS) of universal adhesive system (UAS) applied in etch-and-rinse (ER) and self-etch (SE) strategies.

**Methods.** One hundred forty-four maxillary anterior sound human teeth were divided into six groups based on the types of surface contaminators: no surface contaminator (control) and experimental groups contaminated with blood, saliva, aluminium chloride (ALC), ferric sulphate (FS), and caries disclosing agent (CDA). Then, each group was further subdivided into two, based on the application strategy of UAS (ER and SE). After applying the adhesive according to the manufacturer’s instructions, and bonding cylindrical composite samples, the SBS was measured. The data were analysed using two-way ANOVA, Tukey’s HSD test and *t* test (*P* < 0.05).

**Results.** The SBS in all contaminated groups, except for CDA, was significantly lower in both ER and SE strategies compared to control group (*P* < 0.05). A comparison between the application strategies revealed that ER and SE were only significantly different in the FS contaminated group (*P* < 0.05).

**Conclusion.** All tested contaminators, except CDA, significantly decreased SBS of UAS in both ER and SE strategies.

## Introduction

In order to achieve a proper bonding, it is necessary to prevent any contamination of the substrate with oral fluids. Blood contamination significantly reduces the bond strength (BS) of self-etch (SE) and etch-and-rinse (ER) adhesive systems.^1,2^ Nonetheless, regarding the effect of salivary contamination, different results have been reported depending on the type of adhesive system.^3-5^

Achieving a proper isolation can be considered a significant problem when it is not possible to use a rubber dam. Recently, it has been suggested the use of haemostatic agents (HAs), in order to control bleeding.^6^ It has been shown that the effects of the HAs on the BS depend on the type of adhesive system. Smear layer removal by the HAs can adversely affect the bonding mechanism of SE adhesive systems.^7,8^ However, the effects of these agents on the BS of ER adhesive systems have been inconsistently reported in the literature.^9,10^

Other possible dentine contaminators are caries disclosing agents (CDAs). Some studies have shown that when CDAs are used before bonding procedures, they have no adverse effects on the BS of ER and SE adhesive systems.^11,12^ However, Singh et al., reported significant reduction in the tensile BS of ER adhesive system to sound and carious affected dentin after application of CDAs.^13^

Recently, new type of adhesive systems, known as universal or multi-mode, have been introduced, which allows the dentist to save time and also easily prepare tooth surface in ER, SE or selective etching strategies. Besides the chemical compounds commonly used in the composition of dental adhesives, universal adhesive systems (UASs) have been supplemented with components such as silane and chlorhexidine to provide broader indications and applications.^14^ The aim was to investigate the effects of different surface contaminators (blood, saliva, aluminium chloride [ALC], ferric sulphate [FS], and CDA) on the shear bond strength (SBS) of UAS applied in ER and SE strategies.

## Methods

For the purpose of this in-vitro study, 144 sound maxillary human anterior teeth, extracted for periodontal reasons, were selected. Teeth were stored in 70% ethyl alcohol for three months until the beginning of the study. After cleaning the teeth surfaces from debris and filth by manual scaling, and rubber cap with prophylaxis paste, the root of teeth were mounted in self-cure acrylic resin cylinders while the crown were out. The dentin of facial surfaces was exposed by means of a number 837LG bur mounted on high-speed handpiece under abundant water spray and was polished with 600-grit silicon carbide paper (SiC) under running water for one minute to produce flat bonding surfaces with uniform smear layers. It should be noted that for each five specimens, a new bur and for each surface, a new SiC paper was used. Then, the samples were divided into six groups (n=24) based on surface contaminations:

1. Group I: without any contamination (control);
2. Group II: One drop of blood samples taken from humans were applied on the teeth by a microbrush and then air-dried from a distance of ten cm for 20 seconds;
3. Group III: One drop of saliva taken from human’s mouth was applied on the teeth by a microbrush and then air-dried from a distance of ten cm for 20 seconds;
4. Group IV: ALC HA was applied on the surface of dentin for two mins, then washed with water, and air-dried for 30 seconds;
5. Group V: FS was applied on the surface of dentin for two mins, then washed with water, an air-dried for 30 seconds;
6. Group VI: one drop of CDA was applied on the surface of dentin according to the manufacturer’s instructions for ten seconds and washed for ten seconds.

Then, samples of each group were subdivided into two subgroups based on the application strategy (ER and SE) of the UAS (All Bond Universal, Bisco, Schaumburg, IL, USA). The samples in the ER subgroup (n=12) were etched with 35% phosphoric acid (Select HV Etch, Bisco, Schaumburg, IL, USA) for 15 seconds, washed for ten seconds, and excess moisture was removed by cotton pellets. Afterwards, the UAS was applied on the samples and light-cured according to manufacturer’s instructions for ten seconds using Demetron A2 light curing unit (Kerr, Scafati, Italy). The samples in the SE subgroup were treated by the same UAS applied on the prepared surface of teeth according to the manufacturer’s instructions and light-cured for ten seconds using same light curing unit.

In the ­next step, transparent vinyl cylinders (four mm height and three mm diameter) were first filled with shade A2 of Gradia Direct composite resin (GC, Tokyo, Japan) and placed on the teeth where the adhesive system applied. The extra composite resin was removed with spatula and light-cured for 60 seconds from each side using the same light curing unit. Then all samples were stored in distilled water (37 °C) for 24 hours. SBS was determined using a Universal Testing Machine (Hounsfield Test Equipment, Model H5KS, Surrey, UK) with a cross-head speed of 0.5 mm/min. The SBS was calculated by dividing the obtained force (in Newton) by the restoration surface area (mm^2^) in MPa.

**Figure 1:**
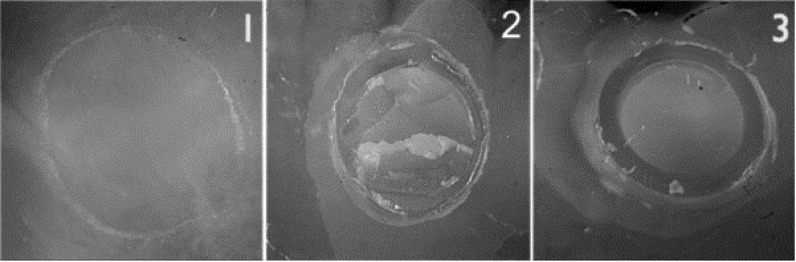
Failure Patterns under Stereomicroscope: 1) Adhesive, 2) Mixed, 3) Cohesive

To determine the pattern of fractures according to the following classification, the samples were examined under a stereomicroscope (magnification ×40) (Figure 1):

1. Adhesive: failure between dentin substrate and repairing composite resin
2. Cohesive: fracture within the repairing composite resin
3. Mixed: a combination of the two above-mentioned patterns

Data were analysed using two-way ANOVA. Tukey’s post-hoc test and *t* test were used for pairwise comparisons at *P* < 0.05.

## Results

Mean ± standard deviations of SBS values and frequency (%) of failure patterns are summarized in Table 1. The Kolmogorov-Smirnov and Shapiro-Wilk tests confirmed normal distribution of data (*P* > 0.05). Levene’s test for the homogeneity of variance showed the establishment of this assumption (*P* > 0.05).

**Table 1:** Mean ± standard deviations (SD) of shear bond strength values (MPa) and frequency (%) of fracture patterns in the study groups

**Adhesive strategy**	**Surface contaminations**	**Shear bond strength (MPa)**		**Fracture patterns (%)**	
		**Mean ± SD**	**Adhesive**	**Cohesive**	**Mixed**
Etch-and-Rinse	Control	29.58 ± 3.81^A^	0 (0%)	4 (33.33%)	8 (66.66%)
	Blood	13.74 ± 3.60^BC^	4 (33.33%)	0 (0%)	8 (66.66%)
	Saliva	15.07 ± 5.47^BC^	5 (41.6%)	0 (0%)	7 (58.4%)
	Aluminium Cl	20.47 ± 5.36^BD^	5 (41.6%)	0 (0%)	7 (58.4%)
	Ferric Sulfate	18.00 ± 3.66^B^	4 (33.33%)	0 (0%)	8 (66.66%)
	Disclosing Agent	25.08 ± 3.60^AD^	4 (33.33%)	4 (33.33%)	4 (33.33%)
Self-etch	Control	28.59 ± 2.27^A^	0 (0%)	3 (25%)	9 (75%)
	Blood	12.32 ± 2.73^B^	5 (41.6%)	0 (0%)	7 (58.4%)
	Saliva	11.53 ± 4.00^B^	3 (25%)	0 (0%)	9 (75%)
	Aluminium Cl	17.74 ± 4.14^B^	7 (58.4%)	0 (0%)	5 (41.6%)
	Ferric Sulfate	13.71 ± 4.17^B^	5 (41.6%)	0 (0%)	7 (58.4%)
	Disclosing Agent	24.82 ± 4.59^A^	4 (33.33%)	4 (33.33%)	4 (33.33%)

Different superscripts represent statistically significant differences (*P* < 0.05).

Two-way ANOVA showed that effect of contamination type (*P* < 0.001) and adhesive application strategy (*P* < 0.001), was statistically significant on the SBS. However, the interaction effect between these variables was not statistically significant (*P* > 0.05).

Tukey’s post hoc test for pairwise comparisons of surface contaminations for both application strategies of the UAS showed that:

- The SBS in the control group significantly differed with all contaminators (*P* < 0.001) except for the CDA (*P* > 0.05).
- The SBS in the blood subgroup revealed significant differences with the ALC and CDA (*P* < 0.05), but it was not significantly different from the saliva and FS (*P* > 0.05).
- The SBS in the saliva subgroup was significantly different from ALC and CDA (*P* < 0.05), but it showed no significant differences with FS (*P* > 0.05).
- The FS and CDA were significantly different (*P* < 0.05). However, in SE strategy the SBS of ALC was significantly different from that of CDA (*P* < 0.001); whereas, in ER strategy there was not any significant difference between these groups (*P* > 0.05).

**Figure 2:**
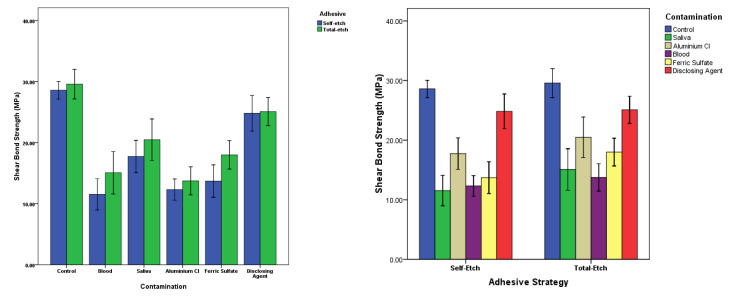
Error-bar graphs of statistical analysis.

Furthermore,a comparison between the adhesive application strategies in various surface contamination groups using *t* test showed that the SBS of the FS in ER mode was significantly different from SE mode (*P* = 0.01). However, no significant differences were observed between the two application strategies in the other types of contaminators (*P* > 0.05). Error-bar graphs are presented in Figure 2 for further clarification.

## Discussion

The tested hypothesis was that different surface contaminations have no effects on the SBS of UAS applied in ER and SE strategies. The findings showed that contamination with saliva, blood, ALC and FS significantly decreased the SBS of UAS in both of application strategies. So, the tested hypothesis was rejected. Similarly, numerous studies have shown that contamination with blood adversely affects the BS.^15,16^ Contamination with blood leads to the formation of a thin layer of macromolecules, such as fibrinogen and blood platelets, on the underlying dentin, which can prevent the penetration of adhesive into the dentinal tubules.^1^ However, Kaneshima et al reported no significant negative effects on the BS with blood contamination before acid etching or prior to applying SE primer; nevertheless, blood contamination after acid etching or applying SE primer resulted in considerable decrease in the BS.^16^

Regarding the effects of saliva contamination, similar to our study, some studies showed that salivary contamination could decrease the BS of SE adhesive systems because either saliva can cause more dilution of acidic primer of the adhesives, or salivary proteins inhibit primer penetration into collagen networks.^1,5^ Furthermore, in the case of ER systems, this may be due to technical complexity so that resin may not penetrate to some demineralized parts of dentin owing to the separate steps of etching and bonding application. Additionally, some parts of demineralized dentin may be contaminated again with saliva after etching and before bonding application.^17,18^ Gupta et al demonstrated that the BS drops more in ER system after saliva contamination in comparison to SE systems, and amount of BS reduction in two-step SE system was significantly more than those in one-step SE system. These may be because one-step SE systems are the least technique sensitive strategy, and SE systems may contain some ingredients such as Phenyl-P or 10-MDP, which improve adhesion to dentin through creation of chemical bonding.^19^

Regarding the effect of ALC contamination, similar to our findings, Ajami et al,^7^ and Kuphasuk et al,^6^ also reported reduced BS of SE adhesives. It has been shown that the use of ALC on the dentinal surface creates varying degrees of demineralization. Removal of the whole smear layer has also been reported after the application of this HA for 5 min. Since the role of the smear layer in the bonding mechanism of SE adhesive systems has been approved, dissolution of this layer after application of ALC adversely influences the BS of these systems.^20^ Even so, contrarily, Kuphasuk et al,^6^ did not report any significant decrease in the BS of ER adhesive system. This may be attributed to the unique chemical composition of UASs, which are quite different and could enhance chemical bonding because of some ingredients such as silane and MDP.

In the case of FS, some previous studies, similarly reported a significant reduction in the BS of SE adhesives.^9,21^ However, contrarily and similar to ALC, some studies failed to show significant decrease in BS of ER systems.^9,21^ FS, besides the ability to demineralize and change the smear layer due to its low pH, is able to coagulate collagen fibres as well as plasma proteins in the dentinal fluid.^22^

Another finding was that, in contrast with all the above contaminators, contamination with CDA had no significant negative effects on the SBS. Similarly, previous investigations presented evidence that contamination with CDA did not decrease the BS, which might be because the smear layer is not removed or disrupt after application of CDA.^11,12^

As another interesting finding, there were no significant differences in the SBS between the two application strategies of UAS in control and blood, saliva, ALC and CDA contaminated groups. This may be attributed to the fact that UAS are quite different from common SE and ER systems in terms of chemical composition. They have unique chemical composition and part of their BS is due to the chemical bonding potential of some of its specific ingredients.^23,24^ However, for FS contamination, SE strategy showed significantly lower BS than that in ER strategy. Furthermore, in SE strategy the BS of FS was significantly lower than ALC. Similarly, O’Keefe et al., demonstrated that the reduction in BS of SE systems following contamination with FS is more than that with ALC.^9^ According to manufacturer’s claim, ViscoStat (FS) is a viscose gel and rinsing with water before bonding procedure is necessary while ViscoStat Clear (ALC) quickly eliminates minor bleeding without forming coagulum or leaving residue adhered to the preparation.

Evaluation of failure patterns showed that cohesive pattern was only observed in the control and CDA groups, while adhesive pattern was not detected in the control group and only belonged to the contaminated groups, which is in agreement with the findings of this study on the reduced BS by different contaminations. The distribution of adhesive and mixed failures in the blood and saliva groups, which are contaminations in oral tissues and contain diverse proteins, were similar to each other but different from in exogenous contaminations such as HAs.

## Conclusion

All surface contaminations studied, except for CDA, significantly reduced SBS of UAS in both ER and SE modes of application compared with the control group. A comparison of the two types of adhesive application strategies showed that SBS in the group contaminated with FS was significantly lower in SE mode than ER mode but other subgroups were not significantly different with respect to the application strategies.

## Authors’ Contributions

This study was planned by MB and MEEC. The literature review was performed by MB, MEEC, SSO and NM. MB and NM performed the experiments. The statistical analyses and interpretation of data were carried out by MEEC and SSO. MB and MEEC drafted the manuscript. All the authors critically revised the manuscript for intellectual con-tent. All the authors have read and approved the final manuscript.

## Acknowledgements

The authors extend their appreciation to the Dental and Periodontal Research Centre at the office of Vice Chancellor for Research and Technology, Tabriz University of Medical Sciences, for the financial support of this research.

## Funding

The study was supported by Dental and Periodontal Re-search Center at Faculty of Dentistry, Tabriz University of Medical Sciences.

## Competing Interests

The authors declare no competing interests with regards to the authorship and/or publication of this article.

## Ethics Approval

This study was approved by the Ethics Committee of the Tabriz University of Medical Sciences (IR.TBZMED.REC.1395.991)

## References

[1] Oztoprak MO, Isik F, Sayinsu K, Arun T, Aydemir B (2007). Effect of blood and saliva contamination on shear bond strength of brackets bonded with 4 adhesives. Am J Orthod Dentofacial Orthop.

[2] Yoo HM, Pereira PN (2006). Effect of blood contamination with 1-step self-etching adhesives on microtensile bond strength to dentin. Oper Dent.

[3] El-Kalla IH, Garcia-Godoy F (1999). Effect of saliva contamination on micromorphological adaptation of single-bottle adhesives to etched enamel. J Clin Pediatr Dent.

[4] Justin RM, Paranthaman H, Rajesh AG, Varghese RP, Ranganath LM (2012). Effect of salivary contamination on the bond strength of total-etch and self-etch adhesive systems: an in vitro study. J Contemp Dent Pract.

[5] Park JW, Lee KC (2004). The influence of salivary contamination on shear bond strength of dentin adhesive systems. Oper Dent.

[6] Kuphasuk W, Harnirattisai C, Senawongse P, Tagami J (2007). Bond strengths of two adhesive systems to dentin contaminated with a hemostatic agent. Oper Dent.

[7] Ajami AA, Kahnamoii MA, Kimyai S, Oskoee SS, Pournaghi-Azar F, Bahari M (2013). Effect of three different contamination removal methods on bond strength of a self-etching adhesive to dentin contaminated with an aluminum chloride hemostatic agent. J Contemp Dent Pract.

[8] Khoroushi M, Hosseini-Shirazi M, Farahbod F, Keshani F (2016). Composite resin bond strength to caries-affected dentin contaminated with 3 different hemostatic agents. Gen Dent.

[9] O’Keefe KL, Pinzon LM, Rivera B, Powers JM (2005). Bond strength of composite to astringent-contaminated dentin using self-etching adhesives. Am J Dent.

[10] Kimmes NS, Olson TL, Shaddy RS, Latta MA (2006). Effect of ViscoStat and ViscoStat Plus on composite shear bond strength in the presence and absence of blood. J Adhes Dent.

[11] Kazemi RB, Meiers JC, Peppers K (2002). Effect of caries disclosing agents on bond strengths of total-etch and self-etching primer dentin bonding systems to resin composite. Oper Dent.

[12] El-Housseiny AA, Jamjoum H (2000). The effect of caries detector dyes and a cavity cleansing agent on composite resin bonding to enamel and dentin. J Clin Pediatr Dent.

[13] Singh UP, Tikku A, Chandra A, Loomba K, Boruah LC (2011). Influence of caries detection dye on bond strength of sound and carious affected dentin: an in-vitro study. J Conserv Dent.

[14] Sofan E, Sofan A, Palaia G, Tenore G, Romeo U, Migliau G (2017). Classification review of dental adhesive systems: from the IV generation to the universal type. Ann Stomatol (Roma).

[15] Chang SW, Cho BH, Lim RY, Kyung SH, Park DS, Oh TS (2010). Effects of blood contamination on microtensile bond strength to dentin of three self-etch adhesives. Oper Dent.

[16] Kaneshima T, Yatani H, Kasai T, Watanabe EK, Yamashita A (2000). The influence of blood contamination on bond strengths between dentin and an adhesive resin cement. Oper Dent.

[17] Sattabanasuk V, Shimada Y, Tagami J (2006). Effects of saliva contamination on dentin bond strength using all-in-one adhesives. J Adhes Dent.

[18] Townsend RD, Dunn WJ (2004). The effect of saliva contamination on enamel and dentin using a self-etching adhesive. J Am Dent Assoc.

[19] Gupta N, Tripathi AM, Saha S, Dhinsa K, Garg A (2015). Effect of saliva on the tensile bond strength of different generation adhesive systems: an in-vitro study. J Clin Diagn Res.

[20] Koibuchi H, Yasuda N, Nakabayashi N (2001). Bonding to dentin with a self-etching primer: the effect of smear layers. Dent Mater.

[21] Ebrahimi SF, Shadman N, Abrishami A (2013). Effect of ferric sulfate contamination on the bonding effectiveness of etch-and-rinse and self-etch adhesives to superficial dentin. J Conserv Dent.

[22] Prabhakar AR, Bedi S (2008). Effect of glutaraldehyde and ferric sulfate on shear bond strength of adhesives to primary dentin. J Indian Soc Pedod Prev Dent.

[23] Wang R, Shi Y, Li T, Pan Y, Cui Y, Xia W (2017). Adhesive interfacial characteristics and the related bonding performance of four self-etching adhesives with different functional monomers applied to dentin. J Dent.

[24] Cardenas AM, Siqueira F, Hass V, Malaquias P, Gutierrez MF, Reis A (2017). Effect of MDP-containing silane and adhesive used alone or in combination on the long-term bond strength and chemical interaction with lithium disilicate ceramics. J Adhes Dent.

